# Does National Context Matter When Women Surpass Their Partner in Status?

**DOI:** 10.3389/fpsyg.2021.670439

**Published:** 2022-02-16

**Authors:** Melissa Vink, Tanja van der Lippe, Belle Derks, Naomi Ellemers

**Affiliations:** ^1^Department of Social, Health, and Organizational Psychology, Utrecht University, Utrecht, Netherlands; ^2^Department of Sociology, Utrecht University, Utrecht, Netherlands

**Keywords:** close relationships, socio-economic status, relationship outcomes, national context, gender stereotypes

## Abstract

There is growing evidence that couples in non-traditional relationships in which the woman attains higher status than her male partner experience more negative relationship outcomes than traditional couples. A possible reason is that non-traditional couples violate persisting gender stereotypes that prescribe men to be breadwinners and women to be caregivers of the family. In the current study (*N* = 2,748), we investigated whether a country’s gender-stereotypical culture predicts non-traditional men and women’s relationship and life outcomes. We used the European Sustainable Workforce Survey, which is conducted in nine European countries. Two indicators of countries’ gender-stereotypical culture are used: Gender Empowerment Measure and implicit gender stereotypes. We found that women’s income and -to a lesser extent- education degree relative to their male partner affected outcomes such as relationship quality, negative emotions, and experienced time pressure. Furthermore, men and women living in countries with a traditional gender-stereotypical culture (e.g., Netherlands, Hungary) reported lower relationship quality when women earned more than their partners. Relative income differences did not affect the relationship quality of participants living in egalitarian countries (e.g., Sweden, Finland). Also, couples in which the woman is more highly educated than the man reported higher relationship quality in egalitarian countries, but not in traditional countries. Our findings suggest that dominant beliefs and ideologies in society can hinder or facilitate couples in non-traditional relationships.

## Introduction

Non-traditional relationships in which women attain higher societal status than their male partners become more common (Pew Research Center, 2013; Portegijs and Van den Brakel, 2018). In almost all western countries, it is nowadays more likely for women to be more highly educated than their male partners (De Hauw et al., 2017). A relationship in which the woman earns more than the man has become more common in recent years (e.g., almost 12% of Dutch women with young children had a higher income than their male partner in 2018 compared to 7% of Dutch women in 2007; Portegijs and Van den Brakel, 2018).

However, non-traditional couples face social and economic penalties as they are perceived more negatively by others (Hettinger et al., 2014; MacInnis and Buliga, 2019; Vink et al., 2021b). Non-traditional couples themselves experience more negative relationship outcomes than couples in traditional relationships (Vink et al., 2021b). When the woman earns more than her husband, both partners tend to be less satisfied with their marriage (Wilcox and Nock, 2006; Bertrand et al., 2015; Zhang, 2015; Syrda, 2019). Moreover, women who work more hours than their male partners report lower relationship quality than women in more traditional relationships (Gong, 2007). Some studies even show that marriages in which the woman is more highly educated than the man are at greater risk of divorce than marriages in which the man is more highly educated (Kalmijn, 2003; Müller, 2003; Goldstein and Harknett, 2006).

It remains unclear why non-traditional couples experience more negative relationship outcomes than traditional couples. Some scholars seek explanations in evolved and universal differences between men and women. They argue that women in general desire partners with good providing skills (e.g., men with high earning potential), whereas men desire partners with good nurturing skills (Buss, 2011). Others argue that it is economically rational if the man is the one who brings home the bacon due to persisting gender inequality in the labor market (Molm and Cook, 1995). However, the differences between men and women are not so stable and are dependent upon the context that they operate in Ellemers (2018). For example, partner preferences are less traditional in countries with a more gender-egalitarian culture (Zentner and Eagly, 2015). Also, couples often fail to make economically rational choices (e.g., women still do the brunt of household tasks, even if they earn more than their male partner; Bittman et al., 2003).

Following this reasoning, we propose that it is more difficult for couples to thrive in a non-traditional relationship in countries with a more traditional gender-stereotypical culture. We define the gender-stereotypical culture as the extent to which social policies and societal norms endorse gender stereotypes, prescribing men to be the breadwinner and women to be the family’s primary caregiver. In the current study, we investigate how a country’s gender-stereotypical culture is related to relationship outcomes of men and women in relationships in which the woman has higher societal status than her male partner. We add to the existing literature by showing that sociocultural factors at the country level have an important influence on men and women’s relationship outcomes in non-traditional relationships.

### The Impact of Gender Stereotypes on the Partner in Close Relationships

In order to understand how social policies and societal norms affect countries’ gender-stereotypical culture, we first describe how gender stereotypes operate. Cultural norms and expectations dictate suitable characteristics and behaviors for both men and women (Eagly et al., 2000; Heilman, 2001; Prentice and Carranza, 2002). Gender stereotypes follow from observing men and women in typical social roles, such as breadwinning men and caregiving women (Social Role Theory; Eagly, 1987; Eagly et al., 2000). People also expect men to take on higher status roles, whereas they expect women to take on lower status roles (Rudman et al., 2012). Men and women who violate gender stereotypes prescribing that the man should have the higher status role within the relationship are at risk of social penalties (Hettinger et al., 2014; MacInnis and Buliga, 2019; Vink et al., 2021b). Others outside the relationships expect a woman with a higher status profession than her male partner to be the dominant one in their relationship and therefore dislike her (i.e., dominance penalty). Also, they expect a man with lower status than his partner to be the weak one in their relationship and therefore disrespect him (i.e., weakness penalty; Vink et al., 2021b). People expect such non-traditional relationships to be less satisfying for the couple than more traditional relationships (Hettinger et al., 2014; Vink et al., 2021b).

Gender stereotypes also have their impact on partners in close relationships who violate gendered status expectations. Women who perceive to have higher societal status than their male partner perceive him to be the weak one in the relationship and, as a result, report lower relationship satisfaction (Vink et al., 2021b). Also, men’s feelings of masculinity were reduced when they interacted with a potential romantic female partner who outsmarted them because gender stereotypes describe men to be intelligent (Prentice and Carranza, 2002; Park et al., 2015). In a similar vein, men’s implicit self-esteem suffers, and men are less optimistic about the future of their relationship when their female partner experiences a success that is more relevant to them (academic success vs. social success; Ratliff and Oishi, 2013). This evidence suggests that non-traditional couples are susceptible to stereotypical expectations in their environment and experience negative relationship outcomes due to these expectations.

Following these difficulties, it comes as no surprise that people prefer to avoid gender role violations (Amanatullah and Morris, 2010; Wallen et al., 2017). Moreover, in reaction to perceived gender role violations, people adhere even more to prescriptive gender stereotypes (Bosson et al., 2009; Willer et al., 2013; Cheryan et al., 2015). Men and women who try to break gender stereotypes thus face a vicious cycle. In order to deal with the difficulties that non-traditional couples experience, it seems more effective to understand and tackle gender stereotypes. This line of reasoning is in line with recent calls of researchers and practitioners to consider the broader system in dealing with gender stereotypes rather than focusing on the individual (Barker et al., 2010). The national culture is one of these larger systems that impact couples’ decisions, behaviors, and feelings through its social policies and through the norms that are endorsed (e.g., Gerson, 1993; Ridgeway and Correll, 2000; Hook, 2006; Payne et al., 2017).

### The Role of National Context

The gender-stereotypical culture of a country determines the extent to which a male breadwinner model is endorsed (Hook, 2006). It plays a crucial role in determining whether attitudes about status divisions within relationships will change (Gerson, 1993). In other words, the gender-stereotypical culture can make it easier or harder for men and women in non-traditional couples to thrive in their relationships. When social policies of a country strongly endorse the male breadwinner model, it is economically less beneficial for couples to break with this model compared to countries that have moved away from the male breadwinner model (Hook, 2006). For example, the state’s universal childcare is associated with women’s full-time labor participation (Gornick et al., 1997). Also, Germany’s social policies have long reinforced the male breadwinner model, whereas, in the United States, social policies less strongly endorse the male breadwinner model (Cooke, 2006). Subsequently, married men who do a larger share of the household work are more likely to divorce in Germany than married men in the United States (Cooke, 2006).

However, the decisions and behaviors of couples cannot be fully understood by economic and practical considerations. In countries that dissuade the male breadwinner norm, women still do most household and childcare-related tasks even if they earn more than their partner (Brines, 1994; Greenstein, 2000; Bittman et al., 2003). The gender-stereotypical culture influences the decisions and behaviors of non-traditional couples both *via* practical and economic considerations as well as *via* considerations of societal expectations (West and Zimmerman, 1987; Gerson, 1993; Hook, 2006). Societal expectations are reflected in country-level implicit gender stereotypes, which also affect the outcomes of people living in such countries (Greenwald et al., 2009; Nosek et al., 2009; Payne et al., 2017). To illustrate, in countries where people held stronger traditional gender associations, larger gender differences in math scores and achievement gaps between men and women in science were found (Nosek et al., 2009).

Following this line of reasoning, we will investigate how the national context affects non-traditional couples’ outcomes by distinguishing two proxies for the gender-stereotypical culture in a country. That is the representation of women in non-stereotypical positions (characterized by the United Nations’ Gender Empowerment Measure, GEM index) and the endorsement of implicit gender stereotypes (characterized by countries’ average scores on the Gender-Career Implicit Association Task, IAT; Greenwald et al., 1998). The country’s representation of women in counter stereotypical positions and its average implicit gender stereotypes define the lives of its inhabitants because they impact the rational and practical decision that couples make (e.g., what status division within the relationship is economically most beneficial?) Furthermore, the representation and salience of implicit gender stereotypes also impact the extent to which couples (unconsciously) anticipate negative social evaluations when they violate traditional gender norms. By including women’s representation in senior positions as well as average country scores on the gender-career implicit association task, we can investigate how these two relevant proxies for the gender-stereotypical culture of a country influence relationship dynamics of men and women in non-traditional relationships. Following Hook (2006), we expect that both women’s representation and average implicit gender associations will affect non-traditional couples in a similar (but not identical) way. By combining these two proxies for a country’s gender-stereotypical culture, we aim to explain a significant amount of variance in non-traditional couples’ experiences in different countries.

### Overview of Study

In the current study, we will investigate how a country’s gender-stereotypical culture affects men and women’s relationship quality, satisfaction with their combination of work and family duties, experienced time pressure, and negative emotions. Relationship quality is an essential predictor of couples’ commitment to their relationship, which predicts the relationship’s persistence (Rusbult et al., 1998). Previous work has shown that women in non-traditional relationships experience decreased work-life satisfaction, more work-life conflict, and emotions such as guilt compared to women in traditional relationships (Vink et al., 2021a). By including work-life satisfaction, experienced time pressure, and negative emotions in this study, we can investigate how having a non-traditional relationship is related to these more individual life outcomes of both men and women. Furthermore, rather than including one objective indicator of the non-traditionality of a relationship, we will include three objective indicators: women’s relative income, educational degree, and working hours in relation to her male partner. Previous work has established that status asymmetry can have negative consequences, now we can establish which indicator is leading.

We will operationalize a country’s gender-stereotypical culture by including an indicator of the endorsement of traditional norms by inhabitants of a country (i.e., the average score on Implicit Association Task per country; IAT-score) as well as an indicator of real gender equality outcomes (i.e., women’s representation in senior positions; GEM index). The IAT is a measure most often used in psychological research, whereas the GEM is often used in sociological research. Using both measures as indicators of a country’s gender-stereotypical culture provides a unique way to combine psychological and sociological measures.

### Hypotheses

In the present research, we will examine whether men and women in non-traditional relationships experience lower relationship and life outcomes than men and women in more traditional relationships. Furthermore, we will study whether the negative outcomes of being in a non-traditional relationship are qualified by gender empowerment and the endorsement of implicit gender stereotypes in the country that men and women live in. Specifically, we will test two pre-registered hypotheses:

*H1*: The higher women’s status relative to their male partner (i.e., the higher women’s relative income, educational degree, and working hours relative to their partner), the more negative relationship- and life-outcomes men and women report.

*H2*: Men and women in relationships in which women have higher status relative to their male partners who live in a country with less gender empowerment and more traditional implicit gender stereotypes will experience worse outcomes compared to men and women in relationships in which women have higher status relative to their male partners and who live in a gender-egalitarian country.

## Materials and Methods

### Participants and Design

To test our hypotheses, we used the European Sustainable Workforce Survey (ESWS; Van der Lippe et al., 2016). The ESWS is a multi-actor organizational survey conducted among employees in nine different countries; Bulgaria, Finland, Germany, Hungary, Netherlands, Portugal, Spain, Sweden, and United Kingdom. The ESWS data is collected in compliance with national, EU, and international ethics-related rules and professional codes of conduct. It has been reviewed by the second author’s Faculty’s Advisory Committee on Ethical Issues, which declared that no ethical approval is necessary. We excluded participants who were not in heterosexual relationships or whose own gender or their partner’s gender was unknown. We excluded participants of whom we were unable to measure their relative income in relation to their total household income from our analyses. These were participants who did not fill out their income or participants of whom we were unable to measure their relative income (e.g., because their own income was higher than the end of the scale of the relative income measure).

Participants (*N* = 2,748 of which 42% men and 58% women; *M*_age_ = 45.03, *SD*_age_ = 10.78) were working in 113 different organizations and had completed a second stage of tertiary education (MA or MSC; 22.2%), upper secondary education (18%) or first stage of tertiary education (BA or BSC; 13.3%). Most participants were married to their partner (71%) and had children living at home (58.7%). Lastly, 12.9% of participants reported being divorced or separated before (see Table 1 for the division of traditional vs. non-traditional couples across countries).

**TABLE 1:** Participant characteristics.

		Total sample%	Male ppn%	Female ppn%	United Kingdom ppn%	German ppn%	Finnish ppn%	Swedish ppn%	Dutch ppn%	Portuguese ppn%	Spanish ppn%	Hungarian ppn%	Bulgarian ppn%
Relative income	Woman earns more	40.7	16.4	58.5	34.3	32.8	24.3	36.3	23.4	31.2	44.1	44.4	56.9
	Woman and man equal	2.7	2.0	3.1	1.9	3.3	10.0	2.8	3.2	1.8	0.0	1.5	2.7
	Man earns more	56.6	81.6	38.4	63.8	63.9	65.7	60.9	73.4	67.0	55.9	54.1	40.4
	*N*	2241	946	1295	105	119	70	220	458	115	118	357	679
Relative education	Woman more highly educated	35.9	28.4	40.6	35.9	23.1	31.9	38.7	29.0	36.3	40.9	38.0	39.3
	Both equally high educated	41.3	42.2	40.7	38.5	47.0	41.7	40.3	44.1	33.3	32.1	39.4	43.1
	Man more highly educated	28.8	28.4	18.7	25.6	29.9	26.4	21.0	26.9	30.4	27.0	22.6	17.6
	*N*	2689	1137	1552	117	134	72	238	501	138	137	452	900
Relative working hours	Woman works more hours	12.4	14.4	10.6	21.2	12.2	18.6	10	8.1	18.1	27.8	10.2	11.5
	Both work equal # of hours	48.7	45.7	51.2	25.3	24.2	32.6	54.7	17.5	51.4	21.1	68.5	70.3
	Man works more hours	38.9	39.9	38.2	53.5	63.6	48.8	35.3	74.7	30.5	51.1	21.3	18.1
	*N*	1890	838	1052	99	99	43	190	383	105	90	305	576

### Procedure

Concerning the ESWS, participants (employees, managers, and the HR manager) were asked to fill out an online or paper-and-pencil questionnaire at their work after the organizations (often HR directors) agreed to participate. The survey took about 20 min to complete. For the current research, we mainly used employee data. The response rate of employees was, on average, 61% (Van der Lippe et al., 2016).

### Materials

#### Demographic Background Information

Participants were asked to indicate their gender, age, marital status (i.e., married vs. cohabiting), whether they were divorced or separated before, and if they had children living at home.

#### Relative Income

To calculate women’s income relative to their male partners, we used participants’ net income in relation to their estimation of their total household income. Net income was asked with the following question: “What are your net monthly earnings from your main job at this organization? Please refer to your average earnings in recent months.” It was explained that net income refers to what participants have left every month after deducting national and local taxes and compulsory national insurance contributions. If participants did not fill out their net income in absolute numbers, they were asked to approximate their net income in 21 categories. These categories were based on a distribution of average income in participants’ own country. To illustrate, Netherlands is a country with a higher average income than Spain. For this reason, the lowest category for participants from Netherlands included all net incomes below 820 euros, whereas this category for Spain included all net incomes below 260 euros. Similarly, the highest category for participants from Netherlands included all incomes above 3,290 euros, whereas this was 2,570 euros for Spanish participants.

Furthermore, participants were asked to indicate their total household income with the following question: “If you combine income from all sources and all household members, which category best describes your household’s total net monthly income?” Participants could choose one of ten categories based on the average household net income per country. We combined participants’ net income with the calculated categories and divided their total household income from participants’ net income per country. To calculate participants’ relative income for each country, we used each category’s means and recoded every answer accordingly. We repeated this procedure for each country and then combined the nine different variables. Lastly, we detracted men’s relative income in relation to their total household income from 1. Thus, our final relative income variable indicated the percentage of women’s net income of the total household income.

#### Relative Education

To calculate women’s educational degrees relative to their male partner’s educational degree, we detracted the man’s highest completed education from the woman’s highest completed education. Participants’ own and their partner’s educational level were asked with one question: “What is the highest level of education that you/your partner have/has completed?” Answers ranged from 0 (*Not completed primary education*) to 7 (*Doctoral degree, Ph.D.*). Higher scores on the relative education variable thus indicate that the woman is higher educated than the man in the relationship.

#### Relative Working Hours

To calculate women’s working hours relative to their male partner’s working hours, we detracted the man’s working hours from the woman’s working hours. We used participants’ and their partners’ contracted working hours, which was asked with one question: “How many hours a week are you/is your partner contracted to work? Exclude any paid or unpaid overtime.” We excluded answers above 80 h a week from our analyses due to plausibility concerns. Higher scores on the relative working hours variable thus indicate that the woman is working more hours than her male partner.

We decided to include relative *contracted* working hours of participants rather than *actual* working hours because the dataset only contains *actual* working hours of the participants themselves and not for their partners. However, the correlation between contracted working hours and actual working hours for participants themselves was very high (*r* = 0.73, *p* < 0.001).

#### Countries’ Gender-Stereotypical Culture: Implicit Gender Stereotypes

To assess countries’ implicit gender stereotypes, we used data made available by Project Implicit^1^ (Greenwald et al., 1998; Nosek et al., 2009). Data were collected among visitors of the Project Implicit website who received educational feedback on social attitudes and stereotypes after participating in an Implicit Association Task. We used the Gender-Career IAT data between 2014 and 2018 and selected scores of participants living in one of the nine countries included in the ESWS (data available^2^). The Gender-Career IAT measures respondents’ association strength of the groups: *men* (e.g., Paul, John) and *women* (e.g., Emily, Anna) with the concepts: *career* (e.g., career, salary) and *family* (e.g., home, children). The IAT consists of two compatible blocks, where respondents were to link the career-words to the male names and family-words to the female names, and two incompatible blocks, where respondents were to link the career-words to the female names and the family-words to the male names. The two compatible and two incompatible blocks were counterbalanced. There were three practice trials. D-scores were calculated by subtracting response latencies of incompatible blocks from compatible blocks and dividing the mean differences in latencies by respondents’ standard deviation on all trials except for the three practice trials. This way, higher scores reflect more traditional implicit associations, and scores close to zero reflect more egalitarian implicit associations (Greenwald et al., 1998). Average D-Scores per country are shown in Table 2.

**TABLE 2:** Average D-scores of Gender-Career IAT from 2014–2018, GEM index and combined Z-scores of IAT and GEM (gender-stereotypical culture) for countries included in ESWS.

	Gender-stereotypical culture	IAT D-Score	GEM index
Sweden	1.62	0.322	0.883
Finland	1.29	0.334	0.853
Spain	1.01	0.332	0.776
United Kingdom	0.49	0.357	0.755
- - - - - - - - - - - - - - - - - - - - - - - - - - - - - - - - - - - - - - - - - - -			
Portugal	0.39	0.346	0.681
Germany	0.27	0.384	0.816
Netherlands	0.15	0.397	0.844
Bulgaria	−0.27	0.364	0.595
Hungary	−1.29	0.414	0.560

*Countries below the dotted line were considered traditional countries, and countries above the dotted line were considered egalitarian countries based on the combined z-scores.*

#### Countries’ Gender-Stereotypical Culture: Gender Empowerment

In order to assess countries’ gender empowerment, we used United Nation’s Gender Empowerment Measurement (GEM) index, which is based on four measures: (1) women’s share of legislators in the national parliament, (2) the percentage of female managers, legislators, and senior officials, (3) amount of female employees in professions and (4) the female-to-male wage ratio among full-time employees. The GEM index is argued to measure women’s agency in society and control over political and economic resources (Maume et al., 2018). We used GEM scores as reported by Maume et al. (2018). The GEM ranges from 0 to 1, with higher scores indicating more gender egalitarianism (see Table 2).

#### Countries’ Gender-Stereotypical Culture: Combined Measure

In order to create one variable of countries’ gender-stereotypical culture, we calculated the average z-score of countries’ implicit gender stereotypes and gender empowerment scores (see Table 2). Higher z-scores indicate a more egalitarian gender-stereotypical culture. Based on these scores, Sweden, Finland, Spain, the United Kingdom, and Portugal were classified as egalitarian countries. Germany, Netherlands, Hungary, and Bulgaria were classified as traditional countries (see Table 2).

#### Relationship Quality

Relationship quality was measured with one question of the time competition survey (Van der Lippe and Glebbeek, 2003). This question was; “In general, how satisfied are you with your relationship?” Answers ranged from 1 (*very unsatisfied*) to 10 (*very satisfied*). Relationship quality is a construct that is often measured with a single item (see, e.g., Hardie et al., 2014; Blom and Hewitt, 2019).

#### Work-Life Satisfaction

Work-life satisfaction was measured with one question: “How satisfied are you with the time you spend on paid work vs. the time you spend on other parts of your life?” (Van der Lippe et al., 2016). Answers ranged from 1 (*extremely dissatisfied*) to 10 (*extremely satisfied*).

#### Time Pressure

In order to measure time pressure, participants were asked to indicate how often the following happened to them: “I am under time pressure,” “I wish I had more time for myself,” “I feel I am under time pressure from others,” and “I cannot deal with important things properly due to a lack of time” (α = 0.85; Van der Lippe et al., 2016). Answers ranged from 1 (*always*) to 5 (*seldom*). We recoded scores so that higher scores indicate more time pressure.

#### Negative Emotions

In order to measure negative emotions, participants were asked to indicate how often during the past week: “you felt depressed,” “you felt that everything you did was an effort,” “your sleep was restless,” “you were happy (recoded),” “you felt lonely,” and “you felt sad” (α = 0.80; Van der Lippe et al., 2016). Answers ranged from 1 (*never*) to 5 (*all the time*).

## Results

### Preliminary Analyses

First, we conducted a correlational analysis to investigate whether background variables were associated with our independent and dependent variables (see Table 3). Participants’ age, marital status, and whether they had children living at home were all associated with several outcome variables. For instance, older participants reported lower relationship quality but higher work-life satisfaction. We included these variables as covariates in our multilevel models. Furthermore, we included participants’ total household income as another covariate to our models. We did this to show that the effects of income, education, and working hours are indeed due to women’s relative position compared to her partner and not because of absolute differences (e.g., couples with higher income in general compared to couples with lower income).

**TABLE 3:** Correlation analyses of background, independent, and dependent variables.

	1	2	3	4	5	6	7	8	9	10	11	12	13	14	15	16	17	18	19	20	21	22
1. Age	–																					
2. Age partner	0.86**	–																				
3. Gender	−0.07**	0.17**	–																			
4. Marital status	0.32**	0.31**	–0.03	–																		
5. Divorced before	0.13**	0.07**	–0.03	−0.27**	–																	
6. Children living at home	0.10**	0.12**	0.04	−0.20**	0.05*	–																
7. Own education level	−0.14**	−0.11**	0.15**	–0.02	−0.08**	–0.01	–															
8. Partner’s education level	−0.12**	−0.13**	–0.02	–0.02	–0.04	–0.03	0.59**	–														
9. Working hours	−0.06**	−0.10**	−0.18**	−0.05*	0.04	0.02	–0.03	0.00	–													
10. Partner’s working hours	−0.06*	–0.01	0.22**	−0.05*	0.04	0.09**	0.01	0.02	0.25**	–												
11. Net income	0.06*	0.04	–0.04	0.01	0.03	0.02	−0.06**	–0.04	0.15**	0.14**	–											
12. Total household income	–0.04	−0.05**	−0.05**	0.06**	0.01	−0.07**	0.31**	0.36**	0.07**	0.05*	0.19**	–										
13. Relative income	–0.01	0.11**	0.49**	−0.06**	0.02	0.06**	0.17**	0.07**	0.01	0.17**	0.01	–0.03	–									
14. Relative education	−0.08**	−0.05*	0.13**	–0.01	–0.02	–0.01	0.03	−0.14**	0.03	0.09**	0.01	–0.02	0.22**	–								
15. Relative working hours	0.01	0.01	0.04	–0.04	0.02	0.12**	0.08**	0.10**	0.12**	0.09**	0.10**	0.06*	0.23**	0.05*	–							
16. Countries’ IAT-score	–0.02	–0.02	0.00	–0.03	–0.02	0.05**	−0.09**	–0.03	–0.03	−0.06**	0.53**	0.05**	–0.04	−0.04*	−0.12**	–						
17. Countries’ GEM-index	–0.02	−0.06**	−0.18**	–0.02	0.04*	–0.04	0.00	0.06**	−0.08**	−0.21**	−0.44**	0.18**	−0.18**	−0.09**	−0.23**	−0.30**	–					
18. Culture (combined z-scores IAT and GEM)	0.00	–0.03	−0.11**	0.01	0.03	−0.06**	0.05**	0.05**	–0.03	−0.09**	−0.59**	0.08**	−0.09**	–0.03	−0.06**	−0.81**	0.81**	–				
19. Relationship quality	−0.09**	−0.11**	−0.08**	0.02	–0.03	0.05*	–0.02	0.03	–0.01	–0.02	0.04	0.05*	−0.10**	–0.01	–0.01	0.03	0.04	0.01	–			
20. Work-life satisfaction	0.09**	0.06**	–0.03	0.09**	–0.03	−0.04*	−0.05*	−0.06**	−0.09**	–0.03	0.03	0.03	−0.06**	−0.05*	–0.04	0.04	0.06**	0.01	0.15**	–		
21. Time pressure	0.00	0.00	0.00	0.02	–0.01	0.02	–0.02	–0.01	–0.03	–0.01	–0.04	–0.02	0.00	0.03	–0.01	–0.01	0.05*	0.04	0.04*	0.05**	–	
22. Negative emotions	0.04	0.08**	0.15**	0.00	–0.01	0.00	0.01	−0.04*	–0.02	0.07**	−0.10**	−0.19**	0.12**	0.05**	0.06**	−0.14**	−0.18**	–0.02	−0.31**	−0.25**	−0.08**	–

**p < 0.01 and *p < 0.05.

*Gender is dummy-coded with 1 = male and 2 = female; Marital status is dummy-coded with 0 = cohabiting and 1 = married; Children living at home is dummy-coded with 0 = yes and 1 = no.*

*Relative income is the percentage of the woman’s income of the total household income.*

*Relative education is calculated by subtracting the man’s educational level from the educational level of the woman.*

*A similar calculation was conducted for relative working hours.*

*Higher scores thus always indicate a higher relative status of the woman in relation to her male partner.*

Next, to prevent multicollinearity, we compared the correlations of our three independent (i.e., relative income, education, and working hours) and moderating variables (i.e., countries’ gender empowerment and implicit gender stereotypes; see Table 3). None of the correlations between the three independent variables were higher than *r* = 0.50 (which we considered problematic regarding multicollinearity). We aim to investigate whether one of the three objective statuses plays a crucial role in couples’ relationship and life outcomes. For this reason, we prefer to use them as separate variables in our model. However, the correlation between the dummies of gender empowerment and implicit gender stereotypes was φ = 0.43, *p* < 0.001. We aim to show how gender stereotypes in countries contribute to couples’ relationship and life outcomes. For this reason, we decided to create z-scores out of the IAT scores and GEM index per country and calculate the mean between these two z-scores. Based on this mean, we created a dummy variable of traditional countries vs. egalitarian countries (see Table 3). We considered combining the two indicators more optimal than running two separate models as this way we were able to run fewer analyses, preventing multiple comparisons. However, we conducted separate analyses for both indicators of a countries’ gender-stereotypical culture, which did not result in many different patterns in the reported results. The only difference we found was that the interaction of relative education and culture on relationship quality was driven by implicit gender stereotypes per country and not by the gender empowerment index.

### Overview of Multilevel Analyses

We conducted two-level multilevel random intercept regression models in SPSS. All models included organization as a Level 2 variable as participants work in 259 different organizations (i.e., multilevel data). First, we conducted multilevel regression models without any predictors to justify the need for random intercept models. These models indicated that there is an especially high variance on the organization level for work-life satisfaction (25.2%), but also relationship quality (5.2%) and negative emotions (5.0%).

In Model 1, we included background variables (i.e., age, marital status, children living at home, and total household income) and women’s income, education, and working hours relative to their partners. In Model 2, we ran one model with the main effects of countries’ gender-stereotypical culture (mean z-scores of IAT and GEM). In Model 3, we ran one model which added the interaction effects of women’s relative status (income, education, and working hours) and countries’ gender-stereotypical culture (see Supplementary Appendix A for regression coefficients and standard errors of all models). Furthermore, in the case of significant interactions, the full model is analyzed separately for traditional vs. egalitarian countries. In case of significant interactions, we will report the simple slopes for the significant status indicators (*M*-1*SD* and M + 1*SD*). Lastly, the ESWS (Van der Lippe et al., 2016) only includes nine different countries, so it could be that our results are driven by one very influential country. In order to check for influential countries, we conducted nine similar analyses, excluding every country once (the Jackknife procedure; Rodgers, 1999; see Supplementary Appendix B). Furthermore, we tested whether participants’ gender qualified our hypotheses. We reran all models and started with a model that included the main effects of participants’ gender (Model 1). Then, we ran an extra model in which we investigated interaction effects of participants’ gender and the relative status indicators (Model 2), and a model that additionally included all two-way interactions of relative status and culture. Last, we ran a model that tested for a three-way interaction between gender, culture, and the relative status indicators (see Supplementary Appendix C). The reported effects below were not qualified by participants’ gender. However, we found three additional effects of participants’ gender, which we have summarized and shown in the Supplementary Appendix C.

### Does Women’s Higher Relative Status Predict Negative Relationship and Life Outcomes?

In line with Hypothesis 1, participants in relationships in which the woman earns more than her male partner reported lower relationship quality and more negative emotions (see Table 4). Furthermore, participants in relationships in which the woman is higher educated than the man reported more time pressure (see Table 4). However, we found no support for Hypothesis 1 on some of the other variables. There were no associations of relative working hours on our dependent variables (see Table 4). Women’s status relative to their partner was not associated with work-life satisfaction (see Table 4). Also, relative income was not associated with experienced time pressure, and relative education was not associated with relationship quality and negative emotions (see Table 4).

**TABLE 4:** Hierarchical linear regression models of main effects of women’s status relative to their partners on dependent variables (model 1) and of main and interaction effects of women’s relative status and culture on dependent variables (model 3).

	Relationship quality		Work-life satisfaction		Time pressure		Negative emotions	
	*b (SE)*	*P*	*b (SE)*	*p*	*b (SE)*	*p*	*b (SE)*	*p*
**Model 1**								
Relative income	−**1.00 (0.28)**	**< 0.001**	−0.33 (0.29)	0.254	−0.01 (0.15)	0.926	**0.26 (0.09)**	**0.003**
Relative education	0.03 (0.03)	0.446	−0.04 (0.04)	0.281	**0.04 (0.02)**	**0.022**	0.00 (0.01)	0.767
Relative working hours	−0.00 (0.00)	0.950	−0.00 (0.01)	0.415	−0.00 (0.00)	0.227	0.00 (0.00)	0.577
**Model 3**								
Relative income	0.16 (0.62)	0.791	−0.28 (0.66)	0.674	0.06 (0.35)	0.870	**0.50 (0.20)**	**0.012**
Relative education	**0.15 (0.06)**	**0.019**	−0.01 (0.06)	0.878	0.06 (0.03)	0.074	−0.02 (0.02)	0.286
Relative working hours	−0.00 (0.01)	0.698	0.02 (0.01)	0.095	−0.00 (0.01)	0.655	−0.00 (0.00)	0.142
Countries’ gender-stereotypical culture	−0.01 (0.11)	0.917	0.19 (0.16)	0.242	−0.07 (0.06)	0.242	−0.06 (0.05)	0.289
Relative income × culture	−**1.47 (0.69)**	**0.034**	−0.04 (0.73)	0.957	−0.08 (0.39)	0.844	−0.30 (0.23)	0.179
Relative education × culture	−**0.18 (0.07)**	**0.014**	−0.04 (0.08)	0.570	−0.03 (0.04)	0.507	0.03 (0.02)	0.149
Relative working hours × culture	0.01 (0.01)	0.631	−**0.03 (0.01)**	**0.020**	−0.00 (0.01)	0.842	**0.01 (0.00)**	**0.045**

*Bold values represent significant effects.*

### Does Countries’ Gender-Stereotypical Culture Qualify These Results?

In line with Hypothesis 2, we found a significant interaction effect of women’s relative income and countries’ gender-stereotypical culture on participants’ relationship quality (see Table 4). Running the models separately for traditional and egalitarian countries, we found that participants living in traditional countries reported lower relationship quality when they had a relationship in which the woman earns more than her male partner, *b* = −1.30, *SE* = 0.31, *p* < 0.001. This was not the case for participants living in egalitarian countries, *b* = 0.22, *SE* = 0.63, *p* = 0.722. Simple slope analyses showed a marginally significant effect for couples in which the woman earns more than the man, *b* = −0.31, *SE* = 0.19, *p* = 0.097. For these couples, living in a traditional country was associated with lower relationship quality than living in an egalitarian country. Simple slope analyses showed no significant effects for couples in which the man earns more than the woman, *b* = 0.29, *SE* = 0.17, *p* = 0.101. In sum, these analyses show that men’s and women’s relationship quality suffers when the woman earns more than her male partner, but this is only the case when these men and women live in a country where a traditional gender-stereotypical culture is endorsed.

Furthermore, we found a significant interaction effect of women’s relative education level and countries’ gender-stereotypical culture on relationship quality (see Table 4). We found no association of women’s educational level relative to her partner and participants’ relationship quality in traditional countries, *b* = −0.04, *SE* = 0.04, *p* = 0.325. In contrast, in egalitarian countries, participants reported higher relationship quality when they were in a relationship in which the woman is higher educated than the man, *b* = 0.14, *SE* = 0.06, *p* = 0.025. Simple slope analyses showed a marginally significant effect for couples in which the woman is higher educated than the man, *b* = −0.26, *SE* = 0.15, *p* = 0.091. For these couples, living in an egalitarian country is associated with higher relationship quality compared to living in a traditional country. Simple slope analyses showed no effects for couples in which the man is higher educated than the woman, *b* = 0.15, *SE* = 0.18, *p* = 0.414.

We also found a significant interaction effect of women’s relative working hours and countries’ gender-stereotypical culture on work-life satisfaction (see Table 4). However, we found no significant differences of participants living in traditional, *b* = −0.01, *SE* = 0.01, *p* = 0.188, vs. egalitarian countries, *b* = 0.02, *SE* = 0.01, *p* = 0.105. Simple slope analyses showed that participants in a relationship in which the man works more hours than the woman were more satisfied with how they combined work and private life, *b* = 0.47, *SE* = 0.20, *p* = 0.022. In contrast, there was no significant effect of participants in a relationship in which the woman works more hours than the man, *b* = −0.10, *SE* = 0.20, *p* = 0.623.

We also found a significant interaction effect of women’s relative working hours and countries’ gender-stereotypical culture on negative emotions (see Table 4). However, we found no significant differences of participants living in traditional, *b* = 0.00, *SE* = 0.01, *p* = 0.246, vs. egalitarian countries, *b* = −0.01, *SE* = 0.00, *p* = 0.109. Simple slope analyses showed that participants in a relationship in which the man works more hours than the woman experienced less negative emotions, *b* = −0.13, *SE* = 0.07, *p* = 0.049, whereas there was no significant effect of participants in a relationship in which the woman works more hours than the man, *b* = 0.02, *SE* = 0.07, *p* = 0.793.

We found no support for Hypothesis 2 on women’s relative status (i.e., relative income, education, and working hours) and experienced time pressure and negative emotions (see Table 4).

### Were There Influential Countries Driving These Results?

Effects remain quite similar when excluding every country once from the analyses (see Supplementary Appendix B). However, the effect of women’s relative income on experienced negative emotions became non-significant when excluding Bulgaria. The effect of women’s relative education on experienced time pressure became non-significant when excluding Bulgaria. The interaction of women’s relative income and gender-stereotypical culture on relationship quality became marginally significant when excluding Sweden and non-significant when excluding Bulgaria. The significant interaction of women’s relative working hours and gender-stereotypical culture on work-life satisfaction became marginally significant when excluding Sweden and Portugal (see Supplementary Appendix B). The results that change due to the jackknife procedure need to be interpreted with care.

## Discussion

In this paper, we investigated the role of national context on relationship and life outcomes of men and women in relationships in which the woman has surpassed the man in societal status. Furthermore, we investigated whether countries’ gender-stereotypical culture (i.e., gender empowerment and implicit gender stereotypes) qualified men and women’s relationship and life outcomes in non-traditional relationships. We replicate and extend previous work showing first evidence of the difficulties men and women experience when they are in a relationship in which the woman has higher status than the man. Our results suggest that especially women’s income and -to a lesser extent- educational degree relative to their male partner negatively impair relationship and life outcomes. When men and women were in a relationship where the woman earns more than the man, they reported lower relationship quality and experienced more negative emotions. When men and women were in a relationship where the woman is higher educated than the man, they experienced more time pressure. Furthermore, these negative outcomes for non-traditional couples are qualified by the gender-stereotypical culture of a country. The salience of gender inequality in a country was conceptualized by a normative, more implicit indicator (i.e., inhabitants’ average implicit gender stereotypes) and a more explicit indicator (i.e., women’s representation in non-stereotypical roles) of a country’s gender-stereotypical culture. This combination of traditional norms and real outcomes in countries affected the relationship quality of non-traditional couples. Specifically, men and women living in traditional countries reported lower relationship quality when they were in a relationship in which the woman earns more than her partner. On the other hand, participants living in egalitarian countries did not differ in relationship quality regardless of the woman’s relative income. Furthermore, we found that men and women living in egalitarian countries reported higher relationship quality when they were in a relationship in which the woman is more highly educated than the man, whereas this was not the case for men and women living in traditional countries.

It is argued that it becomes more accepted for women to be educated and potentially even higher educated than their partner because these relationships are nowadays more common in most European countries (Schwartz and Han, 2014; De Hauw et al., 2017). For this reason, relationships in which the woman is more highly educated than the man have become more stable than before (Schwartz and Han, 2014). On the other hand, although increasing in frequency, relationships in which the woman earns more than the man are still less common (Portegijs and Van den Brakel, 2018; Van Bavel et al., 2018). People still expect men to be breadwinners of their family, whereas they expect women to be their family’s primary caregiver (Park et al., 2010; Morgenroth and Heilman, 2017). Rather than practical differences such as differences in working hours, it seems that especially symbolic status differences between couples explain negative outcomes for non-traditional couples. To this end, an increasing number of paid hours of women alone is not enough to change gender inequality; changing the culture in society is at least as necessary.

### Limitations and Suggestions for Future Research

The national context seems to have a less direct impact on men and women’s life outcomes (i.e., work-life satisfaction, experienced time pressure, and negative emotions). It could be that the gender-stereotypical culture of a country affects couples more indirectly. To illustrate, many women in Netherlands -a country in this study considered to have a traditional gender-stereotypical culture- work part-time (Portegijs and Van den Brakel, 2018). Dutch women who experienced negative life outcomes due to non-traditional divisions of paid work within their relationship might have already reduced their working hours to overcome these negative outcomes. Consequently, they no longer experience time pressure or work-life dissatisfaction because of violating traditional gender roles but have still adjusted their behavior to match the gender-stereotypical culture. Future longitudinal research is needed to investigate the indirect impact of a country’s gender-stereotypical culture on the life outcomes of non-traditional couples.

A limitation of this research is that there were only nine countries in our dataset. Future research should replicate these effects by including more countries. Rather than including a normative (i.e., implicit gender stereotypes) and factual (i.e., gender empowerment) indicator of countries’ gender-stereotypical culture, future research could investigate the role of a more explicit indicator: the salience of non-traditional relationships in a country. The frequency of non-traditional relationships within a country might also capture more indirect ways in which the gender-stereotypical culture affects non-traditional couples. The countries in which non-traditional couples are least common might also be the countries where many couples have internalized the gender-stereotypical culture and have adjusted their roles in the relationship to fit the male breadwinner model. Next to these country-level characteristics, it could be argued that individual- and community-level characteristics influence couples as well. For instance, women’s own implicit gender stereotypes influence how they cope and behave when they perceive to have surpassed their partner in status (Vink et al., 2021a). Also, divorce rates of marriages in which women are higher educated than their male partners are lower in communities where they are more common than communities with more traditional marriages (Theunis et al., 2018). Based on this, a couple’s social network (i.e., having many friends who are also in non-traditional relationships) or working in an organization in which many women have surpassed their partner in status might buffer the negative relationship outcomes for non-traditional couples. People unconsciously shape their implicit gender associations by seeing men and women in typical roles, and when many couples have atypical gender roles, stereotypical associations also become less traditional (Payne et al., 2017). Furthermore, friends and colleagues might provide social support, which is an important factor predicting individuals’ well-being and outcomes. Therefore, social support might be a buffer for couples who break with traditional prescriptive gender stereotypes (Abendroth et al., 2012). Our results suggest that couples’ decisions should not be seen as a private matter but are rather influenced by societal expectations and norms. Future research could include some of the abovementioned characteristics of the context to investigate how they interact and shape non-traditional couples’ realities.

Some of our findings became less strong when one country was excluded from the analysis, indicating some influential countries in our dataset (Rodgers, 1999). Bulgaria was the most influential country and was also the country with the most traditional gender-stereotypical culture. It could be that the gender-stereotypical culture is most salient for non-traditional couples living in Bulgaria and thus also has the most substantial direct impact on the relationship and life outcomes of men and women. Future research should include more countries and investigate whether the salience of a countries’ gender-stereotypical culture indeed explains these effects.

Lastly, women’s relative societal status within the relationship did not affect how satisfied men and women were with their work-life combination. This finding contrasts with earlier findings showing that women reported lower work-life satisfaction in a diary setting when they perceived to have higher societal status than their partner (Vink et al., 2021a). General work-life satisfaction might be something different from daily work-life satisfaction. General measures often show less variety than daily measures (e.g., general measures are more susceptible to socially desirable responses than daily measures; Ohly et al., 2010). For this reason, it could be that the decreased daily work-life satisfaction that non-traditional couples experience is not reflected in their general work-life satisfaction. Non-traditional couples that have experienced dissatisfaction with their work-life combination for a more extended period might have already adjusted their behavior (e.g., by the woman reducing her work hours; Vink et al., 2021a).

### Implications

This work shows how a countries’ gender-stereotypical culture influences people’s relationship and life outcomes and highlights the importance of a structural rather than an individual approach in tackling gender inequality for close relationships. The salience of traditional gender stereotypes prescribing men to be the breadwinner and women to be the primary caregiver of their family on a national scale influences the relationship quality of men and women who break with these expectations. Specifically, our work shows that men and women in relationships in which the woman earns more than her male partner experience more difficulties than couples in more traditional relationships. Furthermore, this is especially the case in countries that endorse traditional gender attitudes (i.e., Hungary, Bulgaria, Netherlands, Germany) and have fewer women in senior positions (i.e., Portugal, Bulgaria, Hungary). Our findings have implications for evolutionary psychologists who argue that there are universal partner preferences between men and women, such that women in general desire partners with good providing skills, whereas men desire partners with good nurturing skills (e.g., Buss, 2011). Our findings show that these preferences may not be so universal and depend at least to some extent on the social norms and national culture, which is in line with scholars who show how partner preferences are influenced by the extent to which countries endorse gender-egalitarian cultures (Zentner and Eagly, 2015).

If social norms about who should be the breadwinner and who should be the caregiver change, couples in which the woman is the one with higher status in the relationship might experience fewer difficulties. For couples living in egalitarian countries, men and women reported higher relationship quality when they were in a relationship in which the woman is more highly educated than the man. This finding is in line with Schwartz and Han (2014). They state that because relationships in which women are more highly educated than their male partners have become more common, these relationships become more accepted and more stable (Schwartz and Han, 2014). The growing evidence that individual outcomes improve not only from interpersonal and more individual approaches (e.g., couple therapy) but also from structural change is essential information for governments and policymakers who try to improve gender equality within societies.

## Conclusion

We show first evidence that countries’ gender-stereotypical culture influences men and women in relationships in which the woman is the one with the highest status of both partners. It turns out to be a bottleneck when women earn more than their male partners and break with the male breadwinner model. These couples’ difficulties are especially salient in countries that endorse the male breadwinner model and have a traditional gender-stereotypical culture. On the other hand, countries characterized by a more egalitarian gender-stereotypical culture seem to facilitate relationships in which men and women have equal status or women with higher status than their male partners.

## Author’s Note

This study is part of the research program Sustainable Cooperation—Roadmaps to Resilient Societies (SCOOP).

## Data Availability Statement

The data analyzed in this study is subject to the following licenses/restrictions: Data is stored at DANS (Data Archive and Networked Services of the Royal Netherlands Academy of Arts and Sciences) with “Restricted Access.” This means that the data are protected and not directly accessible. However, other researchers can request permission to use the (anonymously) stored data. TL has to approve before access is given. Requests to access these datasets should be directed to TL, t.vanderlippe@uu.nl.

## Ethics Statement

Ethical review and approval was not required for the study on human participants in accordance with the local legislation and institutional requirements. The patients/participants provided their written informed consent to participate in this study.

## Author Contributions

MV, TL, and BD contributed to the conception of the study. MV and BD pre-registered the hypotheses and analyses. TL was responsible for data collection. MV organized the dataset and wrote the first draft of the manuscript. MV and TL performed the statistical analyses. TL, BD, and NE wrote sections of the manuscript. All authors contributed to manuscript revision, read, and approved the submitted version.

## Conflict of Interest

The authors declare that the research was conducted in the absence of any commercial or financial relationships that could be construed as a potential conflict of interest.

## Publisher’s Note

All claims expressed in this article are solely those of the authors and do not necessarily represent those of their affiliated organizations, or those of the publisher, the editors and the reviewers. Any product that may be evaluated in this article, or claim that may be made by its manufacturer, is not guaranteed or endorsed by the publisher.

## Footnotes

1
https://implicit.harvard.edu

2
https://osf.io/y9hiq/
