# Supplementary material for: Does National Context Matter When Women Surpass Their Partner in Status?
Source: Front Psychol. 2022 Feb 16;12:670439. doi: 10.3389/fpsyg.2021.670439 (PMC8888434; doi:10.3389/fpsyg.2021.670439)
Supplement: Supplementary file 1 [file Table_1.pdf]

Appendix A: Regression Coefficients and Standard Errors for all Hierarchical Linear Regression Models

Table 1

Hierarchical Linear Regression Models of Women's Status Relative to Their Partners on Relationship Quality

	**Model 1**	**Model 2**	**Model 3**	**Model 3 Traditional Countries**	**Model 3 Egalitarian Countries**
Relative income	**-1.00 (.28)*****	**-1.00 (.28)*****	.16 (.62)	**-1.30 (.31)*****	.22 (.63)
Relative education	.03 (.03)	.03 (.03)	**.15 (.06)***	-.04 (.04)	**.14 (.06)***
Relative working hours	-.00 (.00)	-.00 (.00)	-.00 (.01)	.00 (.01)	-.01 (.01)
Countries' gender-stereotypical culture		.02 (.11)	-.01 (.11)		
Relative income X Culture			**-1.47 (.69)***		
Relative education X Culture			**-.18 (.07)***		
Relative working hours X Culture			.01 (.01)		

Table 2

Hierarchical Linear Regression Models of Women's Status Relative to Their Partners on Work-Life Satisfaction

	**Model 1**	**Model 2**	**Model 3**	**Model 3 Traditional Countries**	**Model 3 Egalitarian Countries**
Relative income	-.33 (.29)	-.34 (.29)	-.28 (.66)	-.33 (.32)	-.33 (.67)
Relative education	-.04 (.04)	-.04 (.04)	-.01 (.06)	-.05 (.04)	-.01 (.07)
Relative working hours	-.00 (.01)	-.00 (.01)	.02 (.01)†	-.01 (.01)†	.02 (.01)
Countries' gender-stereotypical culture		.18 (.16)	.19 (.16)		
Relative income X Culture			-.04 (.73)		
Relative education X Culture			-.04 (.08)		
Relative working hours X Culture			**-.03 (.01)***		

Table 3

Hierarchical Linear Regression Models of Women's Status Relative to Their Partners on Time Pressure

	**Model 1**	**Model 2**	**Model 3**	**Model 3 Traditional Countries**	**Model 3 Egalitarian Countries**
Relative income	-.01 (.15)	-.01 (.15)	.06 (.35)	-.02 (.17)	.08 (.35)
Relative education	**.04 (.02)***	**.04 (.02)***	.06 (.03)†	.03 (.02)	.06 (.04)†
Relative working hours	-.00 (.00)	-.00 (.00)	-.00 (.01)	-.00 (.00)	-.00 (.01)
Countries' gender-stereotypical culture		-.07 (.06)	-.07 (.06)		
Relative income X Culture			-.08 (.39)		
Relative education X Culture			-.03 (.04)		
Relative working hours X Culture			-.00 (.01)		

Table 4

Hierarchical Linear Regression Models of Women's Status Relative to Their Partners on Negative Emotions

	**Model 1**	**Model 2**	**Model 3**	**Model 3 Traditional Countries**	**Model 3 Egalitarian Countries**
Relative income	**.26 (.09)****	**.27 (.09)****	**.50 (.20)***	.18 (.10)†	**.43 (.21)***
Relative education	.00 (.01)	.00 (.01)	-.02 (.02)	.01 (.01)	-.01 (.02)
Relative working hours	.00 (.00)	.00 (.00)	-.00 (.00)	.00 (.00)	-.01 (.00)
Countries' gender-stereotypical culture		-.05 (.05)	-.06 (.05)		
Relative income X Culture			-.30 (.22)		
Relative education X Culture			.03 (.02)		
Relative working hours X Culture			**.01 (.00)***		

Appendix B: Testing for Influential Countries

Table 5

Jackknife-Procedure to Test for Influential Countries Concerning Model 1

	Relationship Quality			Work-Life Satisfaction			Time Pressure			Negative Emotions		
	**Relative Income**	**Relative Education**	**Relative Work Hrs**	**Relative Income**	**Relative Education**	**Relative Work Hrs**	**Relative Income**	**Relative Education**	**Relative Work Hrs**	**Relative Income**	**Relative Education**	**Relative Work Hrs**
**Model 1**	**-1.00 (.28)*****	.03 (.03)	-.00 (.00)	-.33 (.29)	-.04 (.04)	-.00 (.01)	-.01 (.15)	**.04 (.02)***	-.00 (.00)	**.26 (.09)****	.00 (.01)	.00 (.00)
**Without UK**	**-.99 (.28)*****	.01 (.03)	-.00 (.00)	-.30 (.29)	-.04 (.04)	-.00 (.01)	.02 (.15)	**.04 (.02)***	-.00 (.00)	**.25 (.09)****	.00 (.01)	.00 (.00)
**Without Germany**	**-1.07 (.27)*****	.03 (.03)	.00 (.00)	-.22 (.30)	-.03 (.04)	-.00 (.01)	-.07 (.15)	**.05 (.02)***	-.00 (.00)	**.27 (.09)****	-.00 (.01)	.00 (.00)
**Without Finland**	**-1.01 (.28)*****	.03 (.03)	-.00 (.00)	-.34 (.29)	-.04 (.04)	-.00 (.01)	-.02 (.15)	**.04 (.02)***	-.00 (.00)	**.27 (.09)****	.00 (.01)	.00 (.00)
**Without Sweden**	**-1.01 (.28)*****	.03 (.03)	-.00 (.01)	-.36 (.30)	-.03 (.04)	-.00 (.01)	-.04 (.16)	**.04 (.02)***	-.00 (.00)	**.28 (.09)****	.00 (.01)	.00 (.00)
**Without NL**	**-1.03 (.29)*****	.03 (.04)	.00 (.01)	-.31 (.31)	-.04 (.04)	-.00 (.01)	-.02 (.16)	**.04 (.02)****	-.00 (.00)	**.31 (.09)****	.00 (.01)	.00 (.00)
**Without Portugal**	**-1.01 (.28)*****	.03 (.03)	-.00 (.00)	-.30 (.29)	.03 (.04)	-.01 (.01)	-.01 (.15)	**.04 (.02)***	-.00 (.00)	**.25 (.09)****	.00 (.01)	.00 (.00)
**Without Spain**	**-1.03 (.28)*****	.02 (.03)	.00 (.01)	-.30 (.30)	-.04 (.04)	-.01 (.01)	-.00 (.15)	**.05 (.02)***	-.00 (.00)	**.23 (.09)***	.00 (.01)	.00 (.00)
**Without Hungary**	**-.92 (.30)****	.04 (.03)	.00 (.01)	-.31 (.31)	-.04 (.04)	-.00 (.01)	-.04 (.16)	**.05 (.02)***	-.00 (.00)	**.27 (.10)****	.00 (.01)	.00 (.00)
**Without Bulgaria**	**-.62 (.31)***	.06 (.04)	-.00 (.01)	.63 (.34)†	-.07 (.04)†	-.00 (.01)	.15 (.18)	.03 (.02)	-.00 (.00)	.09 (.11)	.01 (.01)	.00 (.00)

Table 6

Jackknife-Procedure to Test for Influential Countries Concerning the Interactions of Model 3

	Relationship Quality			Work-Life Satisfaction			Time Pressure			Negative Emotions		
	**Relative income X Culture**	**Relative education X Culture**	**Relative working hrs X Culture**	**Relative income X Culture**	**Relative education X Culture**	**Relative working hrs X Culture**	**Relative income X Culture**	**Relative education X Culture**	**Relative working hrs X Culture**	**Relative income X Culture**	**Relative education X Culture**	**Relative working hrs X Culture**
**Model 3**	**-1.47 (.69)***	**-.18 (.07)***	.01 (.01)	-.04 (.73)	-.04 (.08)	**-.03 (.01)***	-.08 (.39)	-.03 (.04)	-.00 (.01)	-.30 (.23)	.03 (.02)	**.01 (.00)***
**Without UK**	**-1.63 (.72)***	**-.16 (.08)***	.01 (.01)	-.30 (.77)	-.05 (.08)	**-.03 (.01)****	-.28 (.41)	-.02 (.04)	-.00 (.01)	-.24 (.24)	.03 (.02)	**.01 (.00)***
**Without Germany**	**-1.55 (.68)***	**-.18 (.07)***	.01 (.01)	.10 (.73)	-.03 (.08)	**-.03 (.01)***	-.15 (.39)	-.02 (.04)	-.00 (.01)	-.29 (.23)	.03 (.02)	**.01 (.00)***
**Without Finland**	**-1.40 (.70)***	**-.19 (.08)***	.01 (.01)	-.02 (.74)	-.04 (.08)	**-.03 (.01)***	-.03 (.39)	-.03 (.04)	-.00 (.01)	-.33 (.23)	.04 (.02)	**.01 (.00)***
**Without Sweden**	-1.47 (.77)†	**-.22 (.08)****	.01 (.01)	.20 (.81)	-.10 (.08)	-.02 (.01)†	-.01 (.43)	-.03 (.04)	-.00 (.01)	-.45 (.25)†	**.05 (.02)***	.01 (.00)
**Without NL**	**-1.50 (.72)***	**-.19 (.08)***	.00 (.01)	.00 (.76)	-.06 (.08)	**-.03 (.01)***	-.09 (.39)	-.03 (.04)	-.00 (.01)	-.26 (.23)	.04 (.02)	**.01 (.00)***
**Without Portugal**	**-1.53 (.72)***	**-.20 (.08)****	.01 (.01)	-.18 (.76)	-.05 (.08)	-.02 (.01)†	-.11 (.41)	-.03 (.04)	.00 (.01)	-.31 (.23)	.04 (.02)†	.01 (.00)
**Without Spain**	**-1.54 (.73)***	**-.19 (.08)***	-.00 (.01)	-.11 (.77)	-.05 (.08)	**-.03 (.01)***	-.14 (.41)	-.05 (.04)	.00 (.01)	-.16 (.24)	.03 (.03)	.01 (.00)†
**Without Hungary**	**-1.48 (.70)***	**-.17 (.07)***	.01 (.01)	.03 (.75)	-.05 (.08)	**-.03 (.01)***	-.12 (.40)	-.02 (.04)	-.00 (.01)	-.32 (.23)	.03 (.02)	.01 (.00)†
**Without Bulgaria**	-1.10 (.69)	**-.15 (.08)***	.00 (.01)	-.39 (.76)	-.10 (.08)	**-.03 (.01)***	.09 (.41)	-.05 (.04)	.00 (.01)	**-.59 (.23)***	.05 (.03)†	**.01 (.00)****

Appendix C: Summary and Tables Including Regression Coefficients and Standard Errors for Hierarchical Linear Regression Models Including Gender of Participants

In order to test the effects of participants' gender, we ran similar mixed models by including the main effects of participants' gender (Model 1), the interaction of gender and the three status indicators (Model 2), the interactions of gender and the status indicators, and the interactions of countries' gender-stereotypical culture and the status indicators (Model 3), and finally, the three-way interactions between gender, culture and status indicators (Model 4).

As shown in Tables 7 to 10, the effects reported in the manuscript were not affected by the participant's gender. However, we additionally found three significant interactions. First, there was an interaction of participants' gender and relative education on relationship quality (see Table 7). Men reported being more satisfied with the relationship when they were more highly educated than their partner, whereas this was not the case for women. Furthermore, there was a significant three-way interaction of participants' gender, relative education, and culture on work-life satisfaction (see Table 8). This interaction showed that men living in traditional countries reported less satisfaction with their work-life balance when they had a partner who had a higher education than they had. Last, we found a significant three-way interaction of relative working hours, culture, and participants' gender on work-life satisfaction (see Table 8). This interaction showed that women living in traditional countries and who work more hours than their partners are especially less satisfied with their work-life balance.

Table 7

Hierarchical Linear Regression Models of Women's Status Relative to Their Partners on Relationship Quality

	**Model 1**	**Model 2**	**Model 2 Male Participants**	**Model 2 Female Participants**	**Model 3**	**Model 4**
Relative income	**-.69 (.30)***	.79 (1.06)	.14 (.46)	**-.95 (.42)***	1.71 (1.17)	2.67 (2.06)
Relative education	.02 (.03)	**.25 (.11)***	**.10 (.05)***	-.05 (.05)	**.31 (.12)****	.38 (.19)†
Relative working hours	-.00 (.01)	-.00 (.02)	-.00 (.01)	-.00 (.01)	-.00 (.02)	-.03 (.03)
Gender	**-.26 (.12)***	**-.30 (.11)****			**-.31 (.11)****	-.31 (.11)**
Countries' gender-stereotypical culture					.00 (.11)	-.03 (.12)
Relative income X Gender		-.94 (.64)			-.84 (.65)	-1.52 (1.31)
Relative education X Gender		**-.15 (.07)***			-.11 (.07)†	-.16 (.13)
Relative working hours X Gender		-.00 (.01)			-.01 (.01)	.02 (.02)
Relative income X Culture					-1.33 (.69)†	-2.68 (.24)
Relative education X Culture					**-.17 (.07)***	.27 (.24)
Relative working hours X Culture					.01 (.01)	.04 (.04)
Relative income X Culture X Gender						.93 (1.47)
Relative education X Culture X Gender						.06 (.15)
Relative working hours X Culture X Gender						-.03 (.02)

Table 8

Hierarchical Linear Regression Models of Women's Status Relative to Their Partners on Work-Life Satisfaction

	**Model 1**	**Model 2**	**Model 3**	**Model 4**	**Model 4 Male Ppn / Egalitarian Countries**	**Model 4 Male Ppn / Traditional Countries**	**Model 4 Female Ppn/ Egalitarian Countries**	**Model 4 Female Ppn / Traditional Countries**
Relative income	-.32 (.32)	-.43 (1.12)	-.23 (1.23)	1.02 (2.17)	-.31 (1.13)	-.63 (.60)	-.68 (.93)	-.08 (.50)
Relative education	-.04 (.04)	-.13 (.11)	-.12 (.12)	.29 (.20)	.09 (.10)	**-.18 (.07)****	-.13 (.09)	.04 (.05)
Relative working hours	-.00 (.01)	.02 (.02)	.04 (.02)	-.01 (.03)	.01 (.02)	.00 (.01)	.03 (.01)†	**-.02 (.01)****
Gender	-.01 (.12)	-.01 (.12)	-.02 (.12)	-.04 (.11)				
Countries' gender-stereotypical culture			.19 (.16)	.12 (.16)				
Relative income X Gender		.09 (.68)	.02 (.68)	-.93 (1.38)				
Relative education X Gender		.06 (.07)	.07 (.07)	-.21 (.13)				
Relative working hours X Gender		-.02 (.01)	-.02 (.01)	.02 (.02)				
Relative income X Culture			-.07 (.73)	-1.90 (2.45)				
Relative education X Culture			-.05 (.08)	-.66 (.25)				
Relative working hours X Culture			**-.03 (.01)***	.05 (.04)				
Relative income X Culture X Gender				1.36 (1.55)				
Relative education X Culture X Gender				**.40 (.16)***				
Relative working hours X Culture X Gender				**-.05 (.02)***				

Table 9

Hierarchical Linear Regression Models of Women's Status Relative to Their Partners on Time Pressure

	**Model 1**	**Model 2**	**Model 3**	**Model 4**
Relative income	-.03 (.17)	.21 (.59)	.18 (.66)	.90 (1.17)
Relative education	**.04 (.02)***	.05 (.06)	.07 (.07)	.20 (.11)
Relative working hours	-.00 (.00)	-.00 (.01)	-.00 (.01)	-.03 (.02)
Gender	.01 (.06)	.01 (.06)	.01 (.06)	.00 (.06)
Countries' gender-stereotypical culture			-.07 (.06)	-.09 (.06)
Relative income X Gender		-.16 (.36)	-.09 (.36)	-.62 (.74)
Relative education X Gender		-.01 (.04)	-.01 (.04)	-.10 (.07)
Relative working hours X Gender		-.00 (.01)	-.00 (.01)	.02 (.01)
Relative income X Culture			-.07 (.39)	-1.07 (1.32)
Relative education X Culture			-.03 (.04)	-.23 (.13)†
Relative working hours X Culture			-.00 (.01)	.03 (.02)†
Relative income X Culture X Gender				.72 (.83)
Relative education X Culture X Gender				.13 (.08)
Relative working hours X Culture X Gender				-.02 (.01)†

Table 10

Hierarchical Linear Regression Models of Women's Status Relative to Their Partners on Negative Emotions

	**Model 1**	**Model 2**	**Model 3**	**Model 4**
Relative income	.06 (.10)	-.46 (.34)	-.24 (.38)	.13 (.42)
Relative education	.00 (.01)	-.01 (.03)	-.03 (.04)	.06 (.04)
Relative working hours	.00 (.00)	.01 (.01)	.00 (.01)	-.00 (.01)
Gender	**.19 (.04)*****	**.20 (.04)*****	**.21 (.04)*****	**.21 (.04)*****
Countries' gender-stereotypical culture			-.06 (.05)	-.06 (.05)
Relative income X Gender		.33 (.21)	.37 (.21)†	.13 (.42)
Relative education X Gender		.01 (.02)	.00 (.02)	.06 (.04)
Relative working hours X Gender		-.00 (.01)	-.00 (.00)	-.00 (.01)
Relative income X Culture			-.37 (.22)†	-.86 (.75)
Relative education X Culture			.04 (.02)†	**.16 (.08)***
Relative working hours X Culture			**.01 (.00)***	.01 (.01)
Relative income X Culture X Gender				.31 (.47)
Relative education X Culture X Gender				-.08 (.05)
Relative working hours X Culture X Gender				-.00 (.01)
